# Environmental considerations in the Swedish building and construction industry: the role of costs, institutional setting, and information

**DOI:** 10.1007/s10901-017-9588-8

**Published:** 2018-01-23

**Authors:** Anders Isaksson, Henrik Linderoth

**Affiliations:** 1 Department of Technology Management and Economics, Chalmers University of Technology, Vera Sandbergs Allé 8, 412 96 Göteborg, Sweden; 2 School of Engineering, Jönköping University, Jönköping, Sweden

**Keywords:** Building and construction industry, Decision making, Environmental considerations, Sustainability, Sweden

## Abstract

Despite increasing evidence that being an environment-friendly company not only benefits the environment but also makes long-term economic sense, the transition to a more sustainable society is extremely slow. This is true of the building and construction industry as well. At a strategic level, environmental issues have received more attention with the establishment of roles such as environmental managers and implementation of advanced environmental management systems. However, adoption has been slow in the absence of a holistic approach to environmental challenges, partly reinforced by a perception that giving more than the legally required level of environmental consideration will only add to costs without corresponding financial benefits. This raises the following question that the study aims to answer: What is the most important factor influencing decision makers’ in adopting environmental considerations? To this end, it analysed questionnaire data collected from decision makers in the Swedish construction industry along with an in-depth case study of a specific building and construction company. The results show that decision makers perceive informational and institutional constraints on the adoption of environmental considerations. Lack of information is perceived as the biggest obstacle to environmental considerations. If information and knowledge about clients’ and end users’ financial benefits from adopting environmental considerations need to be exploited, they have to be supported by contractual forms that discard a short-term focus on the investment costs of a building in favour of a focus on long-term operational and maintenance costs and benefits.

## Introduction

The building and construction industry has major impacts on the environment and biodiversity through resource use and emissions. Therefore, the industry’s efforts to reduce these impacts are very important to minimize our ecological footprints. The Brundtland Report (Brundtland Commission 1987) has highlighted the impact of the construction industry and its importance for sustainable development. According to statistics from the United Nations Environment Programme (UNEP), the building and construction industry accounts for 4% of global energy use, 30% of energy-related greenhouse gas emissions, about 12% of water use, and nearly 40% of waste produced (UNEP 2016); similar figures are found in many other reports (Erlandsson and Borg 2003; Assefa et al. 2007; Pérez-Lombard et al. 2008). Referring to statistics from the European Parliament, Zhao and Magoulès (2012) state that buildings account for 40% of total energy use and 36% of total carbon dioxide emissions.

The building and construction industry has made efforts in recent decades to develop green building practices to guide practitioners (see, e.g. Gluch et al. 2014; Zuo and Zhao 2014; Hagbert and Femenías 2016; Olubunmi et al. 2016). However, despite these efforts, mainstream building practices seem not to have undergone any noticeable changes (Van Bueren and De Jong 2007; Ryghaug and Sørensen 2009; Yang and Yang 2014; Hopkins et al. 2016). Many construction projects are still carried out in the traditional way in which short-term solutions are favoured over long-term ones, and building practices are seldom characterized as innovative in a green manner (Demaid and Quintas 2006; Hwang and Tan 2012; Yang and Yang 2014). At a strategic level, Swedish building and construction companies are engaged in environmental work with specialized personnel, like environmental managers and advanced environmental management systems (see Gluch et al. 2009). However, in practice, only a few measures are targeted, like waste management and environmental activities of an administrative kind, and from a holistic perspective, companies seem to have problems when approaching environmental challenges in order to facilitate development (Gluch et al. 2009). Yang (2012) states that holistic thinking is needed with regard to decision making and innovative solutions in order to achieve increased sustainability, which is mutually beneficial for all stakeholders. Holistic thinking is further emphasized by Hopkins (2015), who stresses the need for educating decision makers on the value of life-cycle analysis, instead of solely focusing on short-term construction costs. However, at the same time, there is a perception of a trade-off between profit and environmental care (Porter and Van der Linde 1995; Figge and Hahn 2012; Chen et al. 2016). Hence, the holistic thinking might be constrained by a bounded rationality perspective where companies are considered as profit maximizers refusing to abandon prevailing business objectives and ways of creating economic value (Walsh 2005; Stieb 2009).

In the building and construction industry, this line of reasoning could, at the first glance, be heavily institutionalized owing to its mode of business. Traditionally, a client asks for tenders from contractors, and the company with the lowest tender gets the contract. This implies that managers in construction firms have very small incentives to choose products and production methods that will make the tender more expensive by, for example, adding more features to the products. This institutionalized mode of organizing and doing business is one reason that support tools, like life-cycle costing and life-cycle assessment, are often perceived by practitioners as cumbersome to use (see Dammann and Elle 2006).

On the other hand, there are indications that increasing pressure from stakeholders on companies to adopt more and better environmental practices will force companies to take environmental issues into greater consideration in their strategic and operational decision making (Aguinis and Glavas 2012; Benn et al. 2014). In addition, there is a growing body of research that shows that higher costs of environmental considerations might be compensated by potential higher revenues. For instance, in a study of the economic effects of investments in environmentally sustainable buildings, Eichholtz et al. (2010) found that environmental considerations were associated with premiums in rents and values. Hence, in this case, it is the client who absorbs the higher costs of taking into account extra environmental considerations. For construction companies bidding for the contract, the need to take into account extra environmental considerations is just a prerequisite for the tender.

Nevertheless, the question can be asked whether decision makers consider only increased costs and perceived lack of demand for green products as the only obstacles to environmental considerations or whether there are other factors that influence decision makers’ options to adopt environmental considerations. Alternatively, decision makers in construction companies might not always look beyond the direct costs of environmental considerations. Hence, there might even be a mismatch between the perceived (often short term) value and the actual (often long term) value of environmental considerations. Stakeholder theory even suggests that the better a firm manages its relationships with various stakeholders, the better will be its financial performance (Freeman 1984; Donaldson and Preston 1995). In the context of the natural environment, stakeholders can pressure firms to adopt proactive environmental practices that improve the firms’ environmental performance. This improved performance can increase organizations’ internal efficiency and external legitimacy which, in turn, can lead to competitive advantage and wealth creation (Hart 1995; Hart and Milstein 2003; Esty and Winston 2009).

According to stakeholder theory, it can even be argued that decision makers who understand the demands of their stakeholders in the long run also will create a positive effect on financial performance (Freeman 1984; Donaldson and Preston 1995). This might not only be a direct effect caused by increased revenues but also an indirect effect through improved internal efficiency and external legitimacy, which can in turn lead to competitive advantage and wealth creation (Hart 1995; Hart and Milstein 2003; Esty and Winston 2009).

Accordingly, the first question that can be raised is what factors have an impact on decisions related to production and purchases of products and materials in a construction company. A central point of departure in this study is that all decisions made with regard to production processes and purchases have more or less environment-related dimensions. Thus, is it merely direct and/or other related costs that have an impact on decision making, or do environmental considerations have any impacts when decisions are made? The next question that arises is what consequences decision makers perceive if environmental considerations are taken into account when decisions are made. Do decision makers merely observe increased costs as a consequence, or are there other perceived consequences? Finally, if decision makers should adopt environmental considerations, what obstacles do they perceive when decisions are made? Based on the questions raised, the purpose of this study is to explore factors shaping options for adopting environmental considerations in decision making. The purpose is pursued by combining the results of quantitative data collection based on questionnaires sent to decision makers in the building and construction industry with qualitative data collection based on a case study of the Swedish building and construction industry.

The remainder of this paper is organized as follows: In Sect. 2, environmental issues in the industry are described. In Sect. 3, the methods for data collection are presented. Thereafter, in Sect. 4, the results from the survey are presented and contextualized with the help of a case study. The results are discussed in Sect. 5, and the conclusions are presented in Sect. 6.

## Obstacles to environmental considerations

At least three general underlying reasons can be found for the inertia in adopting environmental considerations in decision making. The first is costs. In business, there is an underlying assumption of rationality, implying that it should be possible to calculate the benefits of environmental considerations (Porter and Van der Linde 1995; Ambec et al. 2013). Accordingly, the environmental impact can be considered one of the strategic issues (see, e.g. Reinhardt 1998). For example, it can be argued that customers do not demand green products. Adopting an environmental approach above what is legally required would then create a competitive disadvantage for the company (see, e.g. York 2008). Managers who attempt to actualize a morally preferable practice above what is required by law increase the cost of doing business for the organization compared to those who simply comply with the law (Alexander 2007). This behaviour in turn causes unnecessary and avoidable harm to the organization, which violates the manager’s responsibility to create and maintain a viable organization that competes successfully in the marketplace (Alexander 2007). However, companies in industries with high potential for severe environmental damage and costs for preventing damage have stronger incentives to adopt environmental considerations (see, e.g. Bartolomeo et al. 2000).

The second reason for the inertia in adopting environmental considerations in decision making is institutional setting. The potential profit from clean production is not necessarily driven by the same business logic as the potential profitability of clean products (Bossle et al. 2016; Foster and Green 2002). This implies that there is a separation between environment-friendly production processes and environment-friendly products. For example, in the automotive industry, the production of polluting cars can be very clean (Williander 2007), or the transportation of ‘green’ products can be very polluting. In the building and construction industry, it could be claimed that institutional factors, such as procurement processes and governance systems, have shaped the business logic focusing on the production process due to the lowest tender policy among clients. This in turn implies that short-term solutions have been favoured over long-term solutions (see, e.g. Dubois and Gadde 2002; Demaid and Quintas 2006; Isaksson et al. 2009; Jacobsson et al. 2017). That is, a separation of environmental considerations has occurred with regard to the production process and product.

The third reason for the inertia in adopting environmental considerations in decision making is information. In production processes, decision makers might lack information helping them to adopt environmental considerations (Isaksson et al. 2009). For example, Maqsood et al. (2007) and Gluch and Räisänen (2009) reported that one obstacle for green building practices is practitioners’ limited knowledge of environmental issues and limited interest in searching for and acquiring environmental information.

## Methodology

A mixed-method approach was considered appropriate for this explorative study, in which quantitative results from a questionnaire were combined with qualitative research based on a case study. It should also be noted that the study does not claim that any generalizations are possible, as the study is limited by the selection and number of companies included in the survey. Nevertheless, the survey is inspired by the theory of reasoned action (TRA) (Ajzen and Fishbein 1980) and the theory of planned behaviour (TPB; Ajzen 1991). TRA/TPB state that the perception of ‘behavioural intention’ and subsequent behaviour is a function of an individual’s attitude towards the behaviour—in this case, taking environmental consideration into account—thus, the results of the study may serve as a valuable input for further studies on users’ perceptions.

### Qualitative data collection

As a first step in our investigation of environmental considerations in the construction industry, we started with a qualitative approach (Eisenhardt 1989) involving semi-structured interviews and participant observation.

A total of 17 semi-structured interviews were conducted with actors at different hierarchical levels within the construction industry. Interviewees were, for example, the CEO of a company, the head of a regional unit, the head of a business district, site managers, information and communication technology managers, project managers and managers in a research and development department. The selection of a wide variety of respondents allowed different perspectives on the topic under study to be uncovered. That is, opinions could be collected from the strategic, tactical, and operational level, and eventual discrepancies could be uncovered. All interviews conducted varied in length from 1 to 2 h.

Second, an ethnographically inspired study (Hammersley and Atkinson 2007; Corbin and Strauss 2008) of a single partnering project was undertaken. Empirical material was collected through participant observation—comprising attendance of 45 meetings encompassing 80 h in total. The construction project, worth approximately €50 million over a period of 2 years, involved the re-building and expansion of a public multi-activity arena. The existing building contained indoor swimming pools and an arena for indoor sports, such as basketball and handball. The expanded arena would contain an adventure pool, new swimming pool, gym, and bowling ground.

The qualitative data collected were then analysed in order to obtain insights into environmental considerations in the construction industry. The results from this part of the study were later used for quantitative data collection and supported us to construct valid questions and constructs. In the interviews, the main aim was to inquire about three overall topics:

- Which issues affects decision making in the production process?
- How does increased demand for environmental considerations affect building and construction projects in general?
- What are the major obstacles for decision makers in the construction industry to take environmental issues into consideration in their daily decision making?

First, in our questions about which issues affected decision making in the production process, we observed that decisions could be divided into two types: those related to choice of materials and products, and those related to choice of production methods. The main issues found to affect these decisions are listed as follows:

Issues affecting choice of materials and products

- Product quality
- Direct purchase cost
- Work environment
- Long-term costs
- Environmental impact

Issues affecting choice production methods

- Cost
- Quality
- Work environment
- Environmental impact

Second, when asking about the possible effects of increased demand for environmental considerations on building and construction projects in general, we identified the following six themes:

- lack of knowledge for making environmentally friendly choices;
- increased environmental consideration leads to higher/lower costs;
- increased environmental consideration leads to lower quality;
- increased environmental consideration leads to higher time consumption;
- environmentally friendly production creates goodwill for the company; and
- environmentally friendly production is beneficial for the end consumer.

Third, when asking about the major obstacles respondents felt were hindering them from taking environmental issues into consideration in their daily decision making were as follows:

- lack of information and knowledge;
- the competitive situation;
- lack of competence among clients;
- the economic situation;
- the contractual form;
- legislation; and
- internal policies.

### Quantitative data collection

The quantitative data collection tool was a web-based questionnaire sent to decision makers in the construction industry. The primary goal of the survey was to capture individuals’ perceptions of variables affecting decisions in the production process, the consequences of adopting environmental considerations, and perceived obstacles to adopting environmental considerations. The variables were derived from the qualitative data collection and the theoretical framework and included, for example, long- and short-term costs, quality, time, contractual form, and competitive situation. After a process of testing and revising the questionnaire, it was distributed to decision makers in large- and medium-sized construction companies in Sweden. The testing of the questionnaire included checks by colleagues at the business school for ambiguities, phrasing, scale logic, language, and ease of understanding the questions. A number of respondents from the previous case studies were also asked to fill in the questionnaire, followed by an interview on how they perceived the question.

In some cases, we got help from the companies through the previously established relationship in sending out a link to the web survey to their employees; in other cases, we collected contact information for decision makers by screening the web pages of Sweden’s largest construction companies. The target population was limited to ‘white-collar workers’, for example, off-site managers, site managers, supervisors, purchasers, estimators, and project managers (that is, decision makers in various levels).

After the initial distribution of the web-based survey, two reminders were sent out. As part of the questionnaire was distributed indirectly through the company, our estimation of the response rate is partly based on information from the company on how many employees were provided with the link to the survey. The researchers contacted 249 respondents directly and approximately 800 indirectly, obtaining a total sample of 1049 individuals. In total, 297 complete questionnaires were received, with an estimated response rate of approximately 28%. The exact numbers of respondents in the different occupational groups are presented in Table 1.

**Table 1:** Number of respondents in occupational groups and age of respondents

Position	Number	%	% Distribution per age group		
			–30	31–50	51–
Off-site manager	42	14.1	4.8	40.5	54.8
Site manager	100	33.7	7.8	46.1	46.1
Supervisor	62	20.9	23.4	35.9	40.6
Estimator/planner	45	15.2	14.3	36.7	49.0
Purchaser	20	6.7	24.0	40.0	36.0
Design	13	4.4	42.1	31.6	26.3
Accounting/finance	3	1.0	70.0	20.0	10.0
Other	12	4.0	50.0	35.0	15.0
Total	297	100	19.0	39.3	41.7

### Variables used

To measure factors affecting a) choice of materials and products and b) production methods, respondents were asked to respond to a number of statements that were related to factors that could affect their decision making. The factors were derived from the findings of our previous qualitative study. Respondents were asked to rank their answers on a scale from 1 (totally disagree) to 6 (totally agree). Statements for choice of materials and products are listed in Table 2, and statements for production methods are listed in Table 3.

**Table 2:** Perceptions of importance of factors affecting the choice of materials and products

Statement	*N*	Mean	Min	Max	SD	1	2	3	4	5
1. When we choose materials and products, **quality** is the most important factor for our decision	296	4.37	1	6	1.10	1				
2. When we choose materials and products, **direct purchase cost** is the most important factor for our decision	296	4.35	1	6	1.30	0.588**	1			
3. When we choose materials and products, impacts on the **work environment** are the most important factor for our decision	295	4.13	1	6	1.18	0.699**	0.462**	1		
4. When we choose materials and products, **long-term costs** are the most important factor for our decision	297	4.09	1	6	1.29	0.058	− 0.067	− 0.009	1	
5. When we choose materials and products, **environmental impacts** are the most important factor for our decision	296	3.86	1	6	1.30	0.711**	0.562**	0.801**	− 0.080	1

Statements on a scale of 1: totally disagree and 6: totally agree. Columns 1–5 show the Pearson product–moment correlation coefficient. ** Correlation is significant at the 0.01 level (2-tailed)

**Table 3:** Perceptions of importance of factors affecting the choice of production methods

Statement	*N*	Mean	Min	Max	SD	1	2	3	4
1. When we choose production methods, cost is the most important factor for our decision	295	4.97	1	6	0.99	1	0.212**	0.151**	0.148*
2. When we choose production methods, quality is the most important factor for our decision	296	4.60	1	6	0.99	0.212**	1	0.660**	0.616**
3. When we choose production methods, work environment is the most important factor for our decision	297	4.52	1	6	1.09	0.151**	0.660**	1	0.750**
4. When we choose production methods, environmental impact is the most important factor for our decision	294	4.00	1	6	1.20	0.148*	0.616**	0.750**	1

Statements on a scale of 1: totally disagree and 6: totally agree. Columns 1–4 show the Pearson product–moment correlation coefficient. ** is significant at the 0.01 level and * at the 0.05 level (2-tailed)

To measure how an increased demand for environmental considerations might affect building and construction projects in general, respondents were asked to agree or disagree with a number of statements related to this question. The statements were derived from our qualitative study. Respondents were asked to rank their answers on a scale of 1 (totally disagree) to 5 (totally agree). These statements are listed in Table 4.

**Table 4:** Perceptions of how increased demand for environmental considerations impacts building and construction projects in general

Statement	*N*	Mean	Min	Max	SD	1	2	3	4	5	6	7
1. An environmental friendly product creates goodwill for the company	294	4.36	1	5	0.85	1						
2. An environmental friendly product is beneficial for the end consumer	292	4.05	1	5	0.93	0.425**	1					
3. Increased environmental consideration leads to higher costs	292	3.35	1	5	1.01	− 0.038	− 0.112	1				
4. Increased environmental consideration leads to higher time consumption	294	3.03	1	5	0.99	− 0.121*	− 0.009	0.386**	1			
5. We often lack the knowledge to make environmentally friendly choices	296	2.86	1	5	1.06	− 0.030	0.071	0.269**	0.186**	1		
6. Increased environmental consideration leads to lower costs	292	2.50	1	5	0.88	0.110	0.164**	− 0.481**	− 0.115	0.048	1	
7. Increased environmental consideration leads to lower quality	296	2.31	1	5	1.00	− 0.149*	− 0.227**	0.314**	0.336**	0.156**	− 0.049	1

Statements on a scale of 1: totally disagree and 5: totally agree. Columns 1–7 show the Pearson product–moment correlation coefficient. ** is significant at the 0.01 level and * at the 0.05 level (2-tailed)

Finally, to measure major obstacles that the respondents felt were hindering them from taking environmental issues into consideration when they make decisions. Respondents were asked to respond to a series of statements (derived from our qualitative study) about this issue. Statements are listed in Table 5.

**Table 5:** Perceived obstacles to adopting environmental considerations when making decisions

Statement	*N*	Mean	Min	Max	SD	1	2	3	4	5	6	7
1. **Lack of information and knowledge** is an obstacle to adopting environmental considerations when I make decisions	295	2.91	1	4	0.73	1						
2. The **competitive situation** is an obstacle to adopting environmental considerations when I make decisions	295	2.80	1	4	0.812	0.224**	1					
3**. Lack of competence among clients** is an obstacle to adopting environmental considerations when I make decisions	293	2.66	1	4	0.848	0.405**	0.318**	1				
4. The **economic situation** is an obstacle to adopting environmental considerations when I make decisions	293	2.49	1	4	0.842	0.091	0.524**	0.267**	1			
5. The **contractual form** is an obstacle to adopting environmental considerations when I make decisions	294	2.28	1	4	0.804	0.233**	0.465**	0.387**	0.285**			
6. **Legislation** is an obstacle to adopting environmental considerations when I make decisions	280	2.00	1	4	0.645	0.053	0.193**	0.097	0.190**	0.233**	1	
7. **Internal policies** are an obstacle to adopting environmental considerations when I make decisions	289	1.72	1	4	0.679	0.084	0.211**	0.150*	0.104	0.256**	0.417**	1

Statements on a scale of (1) no obstacle, (2) minor obstacle, (3) almost a major obstacle, and (4) a major obstacle. Columns 1–7 show the Pearson product–moment correlation coefficient. ** is significant at the 0.01 level and * at the 0.05 level (2-tailed)

## Results

This section presents the results from the survey. First, this section presents respondents’ perceptions about factors they believe impact decision making for purchases of material and products, and choice of production methods. Thereafter, respondents’ perceptions about consequences of environmental considerations for building and construction projects are presented. Then, this section presents respondents’ perceptions about obstacles they perceive if they have to take environmental considerations into account when they make decisions. Finally, this section presents respondents’ perceptions about the environmental management system.

### Attitudes towards factors influencing decision making

When products and materials are chosen, direct purchase costs and quality are ranked as the most important factors influencing the decision, whereas environmental impacts are ranked fifth by the respondents (see Table 2). However, even if environmental impacts rank lowest, one can argue that respondents’ awareness of environmental considerations is high, because respondents agree more than they disagree that environmental impacts matter. Regardless of whether this belief is true, or whether the score is high because it is politically correct to show environmental consciousness, the average respondent is aware of the need to adopt environmental considerations. However, what cannot be determined is how large a part of the investigated population is not aware of these issues.

In a paired-sample *T* test, no significant differences between quality and direct purchase cost were found, but the difference between direct purchase cost and work environment was significant (*p* = 0.020), as was the difference between long-term costs and environmental impacts (*p* = 0.001). Long-term costs are also the only item that is not significantly correlated with any other item.

When respondents’ perceptions about factors influencing decisions related to production methods were measured, cost was the most important factor, followed by quality (see Table 3). Interestingly, costs were judged more important than quality, although both variables had high scores. Again, environmental impacts had the lowest rank, but as in the case of factors affecting the choice of materials and products, there is an awareness of environmental impacts, because respondents agree more than they disagree that environmental impacts matter.

In a paired-sample *T* test, significant differences between cost and quality (*p* = 0.000) and between work environment and environmental impacts (*p* = 0.000) as an independent variable were found.

Moreover, the ranking of quality and long-term costs might be regarded as predictors for environmental impacts. A linear regression was conducted in order to examine the correlation between environmental impacts, as the dependent variable, and quality and long-term costs. A significant model was obtained (F2,293 = 171,450; *p* < 0.000), adjusted *R*^2^ = 0.536.

By drawing on analysis of the correlation between, on the one hand, the environmental impact factors and, on the other, quality and long-term cost, potential predictors for environmental consciousness might have been identified with regard to purchases of products and materials.

### General perceptions about impacts of environmental considerations

When environmental considerations are adopted, increased goodwill for the company and advantages for the end consumer are perceived as the major positive impacts on a building and construction project (Table 4). However, more studies are necessary to investigate what these benefits could be. Regarding the general impacts of environmental considerations on costs, time consumption, and access to knowledge in a building and construction project, the scores show that respondents do not have distinctive perceptions, despite indications that respondents are surer that adopting environmental considerations implies increased costs rather than decreased costs.

### Perceptions about environmental considerations

In Sect. 4.2, it was asked how demand for increased environmental considerations generally affects a building and construction project. A further relevant question is what obstacles respondents perceive if they have to adopt environmental considerations when they make decisions. Table 5 ranks respondents’ judgements on how different factors affect their opportunities to adopt increased environmental considerations in decision making. The most striking result is that lack of information and knowledge is perceived as the biggest obstacle to adopting environmental considerations for decisions made at the individual level. This can be compared with the general attitudes in Sect. 4.2, in which respondents did not perceive lack of knowledge to make environment-friendly choices in building and construction projects in general. This difference can be interpreted as the result of respondents’ thinking that knowledge and information regarding environmental issues might be available somewhere in the company. However, when respondents themselves should make decisions, this knowledge and information is not available. Moreover, a paired-sample *T* test was conducted, and significant differences were found between all the mean values.

### Context of the results

As shown in the results from the survey, cost and quality considerations were the highest ranked factors when decisions are made about material and product purchases, and production methods. This is not surprising, since operations are organized by projects with set budgets and timelines. Furthermore, costs and reduction in costs have been in greater focus in recent years in the industry. The CEO of the company studied stated in an interview that cost control and cost reductions were his major concern for several reasons. He stated that the major problem in the industry was the increase in building costs in recent years, mainly because increased costs could not be transferred to customers. He argued that a continuous cost increase in the industry would imply that potential investors—private, corporate, and public—would choose to spend their resources on other products and services in which they perceived higher value for their invested money. Therefore, a high-priority goal is to reduce costs. To reach this goal, a number of activities are mentioned in interviews with managers at different levels and in public material presenting the company’s strategies for reducing costs. However, with regard to aspects of sustainability, the CEO stated that the industry is just at the beginning of this process and these issues probably would soon climb higher on the agenda. The manager referred to a published article in the *Harvard Business Review* (Lockwood 2006) and reflected that when a well-known business magazine publishes an article on this topic, it would probably receive higher attention in the short term. Other managers interviewed stated that the industry tried to work proactively with regard to materials and chemicals that could damage health and the environment. For example, the industry has developed the so-called Basta list, which contains materials and chemicals that should not be used. Moreover, this is the most common answer that emerges in interviews to questions asked about environmental issues: removal of chemicals and materials with negative effects on health and the environment from production. In this sense, environmental considerations have been a focus in the production process. However, state regulations and new contractual forms have triggered increased focus on the end product and some alignment between the production process and end product. Due to new state regulations, energy consumption in accomplished buildings has received increased focus. However, some managers also claim that new contractual forms have played a significant role in the attention paid to a building’s energy consumption. For example, the so-called partnering contracts imply that focus has been moved from keeping costs as low as possible to obtain the highest possible value for the invested money. This implies, for example, that the contractor and client discuss different solutions before and during the project process. A target cost is set for the project, and if costs are below the budgeted level, the contractor and client share the gains.

Another strategy to decrease costs is to focus on purchases of products and material abroad. However, from an environmental perspective, this strategy can be seen as a bit contradictory. First, purchases abroad may lead to longer transportation distances and higher emissions of CO_2_. Second, due to varying environmental regulations in different countries, materials purchased abroad may have been produced with higher emissions of polluting substances. However, purchasing abroad is encouraged at all managerial levels. The focus on purchasing abroad is reflected in one of the company’s regional news magazines, which depicts a chart showing the ‘competition’ between business district managers, specifically the proportion of the total value of their purchases that originates from purchases abroad. However, if the company’s focus on reducing costs is taken into consideration, the focus on purchases abroad is just a logical consequence.

In the results of the survey and from interviews with managers, it is found that maintaining and reducing costs have the highest priority in construction projects, but at the same time, there are no clear-cut perceptions that costs always are a major obstacle to environmental considerations. At the same time, the focus on costs is accompanied by a focus on quality.

## Discussion

The aim of the study was to explore factors shaping options for adopting environmental considerations in decision making. This aim was fulfilled by scrutinizing three different issues. (1) What factors have an impact on decisions related to production and purchases of products and materials in a company? (2) What consequences do decision makers perceive if environmental considerations are taken into account when decisions are made? (3) If decision makers have environmental considerations, what obstacles do they perceive when making decisions?

Regarding the factors that have an impact on decisions related to production and purchases of products and materials, the cost—together with quality—was the most important factor. It is possible that quality can be considered a financial factor. One argument for this line of reasoning is that quality controls are aimed at doing right from the beginning, that is, avoiding costly re-works later in a project. Moreover, the scores show that decision makers are aware of environmental impacts, even if this item was ranked the lowest of five items.

This study shows, as earlier writing on the trade-off between profit and environment has done, that financial constraints are not the first obstacle that respondents perceive when decision makers adopt environmental considerations. Surprisingly, lack of information is perceived as the biggest obstacle at the individual level, even if the financial factor was most important when decisions about products, materials, and production processes were made. However, what kind of information is sought? With regard to the highest ranked items influencing choice of material, products, and production processes, it is possible that actors want more knowledge and information about cost and quality of more environment-friendly alternatives. Will costs increase and quality decrease if more environment-friendly alternatives are chosen? Regarding quality, it can be assumed that the ambiguity might not be too high. First, there is a strong correlation between quality and environmental consideration when purchase decisions are made. This result is in line with observations by Bossink (2004), who showed that construction projects aimed at green innovation raised the quality of construction projects. Second, Table 4 shows that respondents do not agree that increased environmental considerations will have a negative impact on quality. However, Table 4 also shows that respondents only moderately agree that environmental considerations imply higher costs, even if the score does not show a distinctive perception. With regard to the perception that there is a trade-off between profit and environment (Chen et al. 2016), backed by the findings from this study, it might be fruitful to study corporate-level perceptions and perceptions at the individual level regarding the relationship between environmental considerations and costs.

Lack of competence among clients was ranked as the third obstacle to adopting environmental considerations in decision making. This item can be connected to the claim that customers do not demand green products, which in the long run would lead to a competitive disadvantage for companies that focus on green products more than its competitors do (see, e.g. York 2008). However, with regard to the construction industry and its clients, it seems that traditional procurement systems have not given the contractor or client any incentives to focus on long-term issues. For example, in a fixed-price contract, finding cheaper solutions has been the contractor’s only way to make a better profit. In this sense, it is possible that institutional factors, that is, established procurement systems, have reinforced a separation between the production process and the final product. That is, environmental considerations can be adopted in the production process, but if a more environment-friendly end product was to require higher investment costs, the contractor would avoid making the bid more costly, because the contractor would then risk losing the tender. However, there are institutional opportunities for adopting increased environmental considerations, the first and foremost of which depends on what kind of contractual form regulates the building and construction project (see also Wenblad 2001). Projects regulated by partnering contracts are one arena for adopting increased environmental considerations, but if this is possible, then better information and knowledge is necessary when decisions are made. In addition, the partnering contract is a window of opportunity for linking decisions made in the production process to attributes of the final product. This means that, for example, energy consumption and other operational and maintenance costs can be taken into consideration to a wider extent than prescribed by standards, at least as long as there is a healthy payback on increased investments. However, this requires that the focus moves from the cost of the investment in a building to the costs of its operation and maintenance. Thus, new contractual forms can open up for dialogue and collaborative learning, which is vital if stakeholders are to engage in environmental issues (see also Kaatz et al. 2006; Mathur et al. 2008). However, in this case (professional) clients need to incentivize this development, because contractors mainly adapt to market conditions (see also Bosch-Sijtsema et al. 2017; Jacobsson et al. 2017; Jacobsson and Linderoth 2010). The first step in this direction may be the increased interest in environmental certifications, for example LEED or BREEAM, of buildings, among clients. This implies that contractors do not always have to analyse trade-offs between costs and the environment with regard to the product. Giving more than the minimum requirement of environmental consideration is instead a prerequisite for the submission of a competitive tender.

## Conclusions

The results show that even if decision makers are aware of environmental issues, cost is the major factor that influences decision makers in the construction industry. However, respondents’ perceptions of how environmental considerations influence costs are not distinctive. Respondents are more inclined to believe that environmental considerations will not decrease costs than that costs will increase as a consequence of environmental considerations. A major finding in this study was that the informational factor, that is, a lack of information and knowledge, was perceived as the biggest obstacle to individuals adopting environmental considerations in their decision making, followed by financial factors (competitive situation), and institutional factors in terms of client competence.

Finally, costs, institutional setting, and information are tightly intertwined with regard to adopting environmental considerations, but at present, they are misaligned. Alignment could be realized if respondents had strong beliefs that adopting environmental considerations would be beneficial for the end consumer, but at the same time, respondents felt that lack of client competence was an obstacle to adopting environmental considerations. Thus, the challenge is to raise awareness among clients by providing evidence that adopting environmental considerations in the production process is beneficial for the end consumer and client. However, the first obstacle relates to information. In order to raise awareness among clients, managers probably do not have the information and knowledge necessary to provide evidence that adopting environmental considerations in the production process is beneficial for the end consumer and client. The second obstacle relates to the institutional setting. Historically, the client has relied on the lowest price tender policy, implying that investment cost has received more attention than long-term costs. This implies that all elements that make a bid more costly have not been included in the bid. If environmental considerations were adopted, appropriate contractual forms would be needed, with a focus on long-term operational and maintenance costs instead of solely on the investment cost for the building.
